# An Investigation of Hierachical Protein Recruitment to the Inhibitory Platelet Receptor, G6B-b

**DOI:** 10.1371/journal.pone.0049543

**Published:** 2012-11-19

**Authors:** Carmen H. Coxon, Amanda J. Sadler, Jiandong Huo, R. Duncan Campbell

**Affiliations:** 1 Department of Physiology, Anatomy and Genetics, University of Oxford, Oxford, United Kingdom; 2 Weatherall Institute of Molecular Medicine, University of Oxford, John Radcliffe Hospital, Headington, Oxford, United Kingdom; Hungarian Academy of Sciences, Hungary

## Abstract

Platelet activation is regulated by both positive and negative signals. G6B-b is an inhibitory platelet receptor with an immunoreceptor tyrosine-based inhibitory motif (ITIM) and an immunoreceptor tyrosine-based switch motif (ITSM). The molecular basis of inhibition by G6B-b is currently unknown but thought to involve the SH2 domain-containing tyrosine phosphatase SHP-1. Here we show that G6B-b also associates with SHP-2, as well as SHP-1, in human platelets. Using a number of biochemical approaches, we found these interactions to be direct and that the tandem SH2 domains of SHP-2 demonstrated a binding affinity for G6B-b 100-fold higher than that of SHP-1. It was also observed that while SHP-1 has an absolute requirement for phosphorylation at both motifs to bind, SHP-2 can associate with G6B-b when only one motif is phosphorylated, with the N-terminal SH2 domain and the ITIM being most important for the interaction. A number of other previously unreported SH2 domain-containing proteins, including Syk and PLCγ2, also demonstrated specificity for G6B-b phosphomotifs and may serve to explain the observation that G6B-b remains inhibitory in the absence of both SHP-1 and SHP-2. In addition, the presence of dual phosphorylated G6B-b in washed human platelets can reduce the EC_50_ for both CRP and collagen.

## Introduction

Platelets are essential for primary haemostasis, but also play a major role in the development of cardiovascular disease. Platelets are regulated by both activating and inhibitory signals and the balance of these opposing signals regulates the extent of cell activation and thrombus formation. Healthy endothelium releases soluble factors, such as prostacyclin (PGI_2_) and nitric oxide (NO), which act to inhibit platelet activation. In addition to these soluble factors, platelets express a number of immunoglobulin (Ig)-like receptors at the platelet surface that relay a negative signal to the cell to either maintain the resting state or limit the extent of activation. These receptors elicit their effects via the immunoreceptor tyrosine-based inhibitory motifs (ITIM) or immunoreceptor tyrosine-based switch motifs (ITSM) in their intracellular tails, which can interact with Src homology (SH) 2 domain-containing proteins. There are a number of Ig-like ITIM/ITSM receptors expressed on the platelet surface, including G6B-b, platelet endothelial cell adhesion molecule-1 (PECAM-1), carcinoembryonic antigen cell adhesion molecule-1 (CEACAM1) and TREM-like transcript-1 (TLT-1).

G6B-b is a major ITIM/ITSM immunoglobulin-like receptor that has been shown to negatively regulate platelet function [1]–[3]. There are an estimated ∼20–25,000 copies of G6B per cell [4], making it one of the most highly expressed platelet cell surface proteins. The receptor is N-glycosylated and migrates as a distinctive doublet at ∼25–28 kDa. Using cross-linking polyclonal antibodies, it has been demonstrated that G6B-b inhibits platelet aggregation in response to collagen-related peptide (CRP) and ADP, and is upregulated at the cell surface 2–4 fold by CRP, ADP and thrombin [1], [3], [5]. G6B-b, has one ITIM and one ITSM in its intracellular tail that preferentially recruit the SH2 domain-containing phosphatases SHP-1 and SHP-2 [1], [3], [6]. Although association of both tyrosine phosphatases has been demonstrated in K562 cells [6], only SHP-1 has been identified as a binding partner for G6B in platelets [1], [3]. While these co-immunopreciption studies demonstrated an association with the receptor, evidence of direct binding has not yet been demonstrated.

ITIM receptors can inhibit signalling from ITAM receptors when co-aggregated, such as that seen with PECAM-1-mediated inhibition of IgE-induced mast cell activation through the recruitment of SHP-2 [7], or FcγRIIB-mediated inhibition of TCR, BCR and FcR signalling [8]–[10]. It is possible that G6B-b may inhibit platelet activation through a similar mechanism, delivering SHP-1, and potentially SHP-2, to the GPVI/FcRγ collagen receptor to oppose signalling from the ITAM. The fact that G6B-b can associate with both tyrosine phosphatases *in vitro* supports such a hypothesis. However, G6B-b has been shown to inhibit GPVI/FcRγ signalling in DT40 cells devoid of both SHP-1 and SHP-2, or the lipid phosphatase, SHIP-1 [11], suggesting that G6B-b may act via an alternative mechanism.

The ligand for this receptor is currently unknown; *in vitro* studies have been hampered by a lack of receptor agonists or antagonists, endogenous or otherwise, with which to study receptor signalling. We know that G6B-b can inhibit CRP- and ADP-induced platelet aggregation [1] and that the receptor can associate with SHP-1 (platelets [1]) and SHP-2 (Cos7 and K562 cells [6]). However, loss of SHP-1 and SHP-2, and also SHIP, does not abolish G6B-b-mediated inhibition of GPVI signalling in DT40 cells [11]. In the current study, we sought to address two questions, the first relating to the biochemical hierarchy of phosphatase recruitment to the receptor, and the second to look for alternative binding partners that may explain the G6B-b-mediated inhibition of GPVI signalling in DT40 cells in the absence of SHP-1, SHP-2 and SHIP-1. To address these questions, we used a combination of *in vitro* biochemical and biophysical techniques to examine phosphatase recruitment to the receptor and also to identify novel binding partners that may give clues to how G6B-b inhibits GPVI signalling in the absence of SH2 domain-containing phosphatases.

## Materials and Methods

### Materials

Biotinylated 60-mer peptides corresponding to amino acids 182 to 241 of G6B-b, were generated by Peptide Synthetics (Hampshire, U.K.). Streptactin-Sepharose beads were purchased from GE Lifesciences (Chalfont, U.K.). The cell permeant SKF inhibitor, PP2, and its inactive analogue, PP3, were purchased from Merck Biosciences. The G6B monoclonal antibody has been previously described [1]. The phosphotyrosine antibody used for Biacore experiments was purchased from Sigma (Poole, UK). The 4G10 anti-phosphotyrosine antibody was purchased from Millipore. All other antibodies were purchased from Santa Cruz Biotechnology (Insight Biotechnology, Wembley, UK). All other materials were purchased from Sigma (Poole, UK).

### Isolation of human platelets

Whole blood was taken from healthy volunteers and collected into 50 ml syringes containing 5 ml acid citrate-dextrose (ACD) in accordance with procedures approved by the Local Research Ethics Committee (Ref: 07/Q1603/17). Platelet-rich plasma (PRP) was isolated by centrifugation at 200 g for 10 minutes at room temperature. PRP was pooled and 10 µg PGI_2_ was added before centrifugation at 1000 g for 10 minutes at room temperature. Platelets were resuspended in 1 ml Tyrodes buffer (134 mM NaCl, 0.34 mM Na_2_HPO_4_, 2.9 mM KCl, 12 mM NaHCO_3_, 20 mM HEPES, 5 mM glucose, 1 mM MgCl_2_, pH 7.3) pre-warmed to 30°C and 150 µl ACD. The volume was adjusted to 25 ml with Tyrodes buffer, followed by 3 ml ACD and 1.25 µg PGI_2_. Cells were centrifuged at 1000 g for 10 minutes at room temperature and the resultant cell pellet was resuspended in 1 ml pre-warmed Tyrodes buffer before adjusting the volume to a final cell density of 4×10^8^ cells/ml (aggregations) or 2×10^9^ cells/ml (peptide pull downs). Where appropriate, 1 mM EGTA, 10 µM indomethacin and 2 U/ml apyrase were added to inhibit aggregations (referred to as non-aggregating conditions).

### Peptide pull down assay

Forty micrograms of peptide were coupled to 50 µl of Streptactin beads (50% slurry) for 30 minutes at 4°C with end-over-end mixing, washed twice with 1 ml PBS and resuspended in 100 µl of modified Tyrodes buffer. Platelets were resuspended in modified Tyrodes buffer at a concentration of 2×10^9^ cells/ml under non-aggregating conditions (10 µM indomethacin, 1 mM EGTA and 2 U/ml apyrase). Cells were activated with 1 ug/ml CRP for 90 seconds under stirring, but non-aggregating conditions, before reactions were terminated by the addition of 500 µl 2× NP40 lysis buffer (150 mM NaCl, 10 mM Tris pH 7.5, 1 mM EGTA, 1 mM EDTA, 1% NP-40) with inhibitors (Roche Complete mini protease inhibitor cocktail, 5 mM NaF, 5 mM NaVO_4_, 250 nM beta-glycerophosphate) and placed on ice. Lysates were pooled and pre-cleared as a batch with 200 µl streptactin beads (50% slurry). Non-specific interactions were then removed by centrifugation at 4,750 g for 15 minutes at 4°C. Pre-cleared lysates were incubated with 50 µl peptide/bead conjugate (50% slurry) at 4°C for 30 minutes with end-over-end mixing. The pre-cleared lysate was divided equally among the samples so that peptide-protein complexes were isolated from the same starting material. Bead/protein precipitates were washed 4 times in 1× NP40 lysis buffer and recovered by boiling in reducing Laemmli buffer prior to separation by SDS-PAGE under denaturing conditions.

### Endogenous immunoprecipitations

One microgram of antibody was bound to 10 µl of Protein A-sepharose (20 µl of a 50% slurry), in a final volume of 100 µl PBS, by incubation on a rotating wheel for 90 minutes at 4°C. Washed human platelets were incubated with either Tyrodes, 5 µM PP2, 5 µM PP3 or pervanadate for 10 minutes, after which they were pelleted and lysed in DP lysis buffer (20 mM Tris pH 7.5, 150 mM NaCl, 5 mM NaOV4, 1× Complete Protease Inhibitor (Roche) and 5% w/v n-Dodecyl-β-D-maltoside (Calbiochem)) such that each IP sample received 4×10^8^ platelets in a final volume of 100 µl DP lysis buffer. Samples were mixed on a rotating wheel at 4°C for 1 hour. Samples were washed once in 200 µl DP lysis buffer and twice in 1 ml PBS prior to boiling in Laemmli buffer for 5 minutes.

### Immunoblotting

Proteins were separated by SDS-PAGE on pre-cast 4–12% NuPage polyacrylamide gels (Invitrogen) under denaturing conditions and transferred onto nitrocellulose membrane. Membranes were blocked with 5% Marvel/TBS/0.1% Tween-20 for 1 hour at room temperature. Primary and secondary antibody HRP conjugates were used according to the manufacturers' instructions. Bands were visualised with ECL Plus reagent (GE Lifesciences, Little Chalfont, U.K).

### Recombinant proteins

Constructs containing SHIP-1 SH2(His-tag) (Oxford Module Consortium [www.omc.ox.ac.uk] construct #3915), SHP-1 (N+C)SH2 domains (maltose binding protein-tag) (OMC #3908M), SHP-2 (N+C)SH2 domains (His-tag) (3911), SHP-1 (N)SH2 (His-tag) (OMC #3909), SHP-1 (C)SH2 (His-tag) (OMC #3910), SHP-2 (N)SH2 (His-tag) (OMC #3912) and SHP-2 (C)SH2 (His-tag) (OMC #3913). These proteins were expressed in BL21(DE3) cells (Invitrogen) induced with 1 mM IPTG for 4 hours at 37°C. Bacteria were pelleted and washed in PBS/0.5 M NaCl prior to sonication in PBS/0.5 M NaCl, in the presence of protease inhibitor cocktail (Complete, EDTA-free, Roche). Soluble and insoluble fractions were separated by centrifugation at 30,000 g for 60 minutes. Soluble fractions containing tagged proteins were incubated for 1 hour at 4°C with end-over-end mixing in the presence of washed Ni-Sepharose (GE Lifesceinces, Little Chalfont, U.K.) supplemented with 40 mM imidazole or Amylose resin (NEB, Herts, U.K). Proteins were eluted according to the manufacturer's instructions and purified by size exclusion chromatography on an AKTApurifier (GE Lifesciences, Little Chalfont, U.K).

### Direct pull down assay

For the tandem SH2 domain assays, 100 pM peptide were immobilised on 25 µl of Streptactin beads (50 µl of a 50% slurry in PBS) (GE Lifesciences, Chalfont, U.K.) and incubated with 100 pM of either SHP-1 SH2 MBP or SHP2 SH2 His protein in a total volume of 250 µl PBS for 30 minutes with end-over-end mixing at room temperature. Beads and supernatant were separated and the beads resuspended in a volume of PBS equal to the supernatant fraction. Equal volumes were loaded onto a 4–12% NuPage gel (Invitrogen) and separated by SDS-PAGE under denaturing conditions. Gels were stained with SimplyBlue Coomassie Stain (Invitrogen). For the single SH2 domain assays, 500 pM peptide was coupled to 25 µl Streptactin beads and incubated with 500 pM purified SH2 domain as described above.

### BIACore analysis

Experiments were conducted on a BIAcore™ 3000 in HBS buffer (0.01 M HEPES, pH 7.4, containing 0.15 M NaCl, 3 mM EDTA, and 0.005% v/v P-20, GE Life Sciences, Amersham, UK). Streptavidin was coupled to a research grade CM5 chip (GE Life Sciences, Amersham, UK) as previously described [12], [13]. Protein-peptide interactions were carried out a rate of 10 µl/min at a constant temperature of 37°C to more accurately reflect physiological conditions. Biotinylated peptides were bound to the streptavidin-coated CM5 chips at 37°C to between 25–50 RU [14]. Purified monomeric SH2 domains were passed over the immobilised peptides at a range of concentrations. Anti-phosphotyrosine monoclonal antibody (Sigma, Poole, U.K.) was passed over the chip to saturation at the end of each experiment to verify peptide coverage on the chip. Binding affinities for the single ITIM/ITSM peptides were determined by plotting equilibrium binding measurements (RU) against SH2 domain protein concentration (Origin 8, OriginLab, MA, USA). K_D_ was defined as the concentration of SH2 protein that gave half maximal binding to the peptide. For the 60-mer peptides, SH2 domains were passed over the chip surface at 50 µl/min. K_D_ was determined as K_off_/K_on_, which were determined using both GraphPad Prism 5.0 (GraphPad, La Jolla, CA, USA) and BIAevaluation Software (GE Life Sciences, Amersham,UK).

### Peptide transduction

Fluorescein-labelled, TAT-G6B-b peptides were synthesised by Peptide Protein Research Ltd (Hampshire, U.K.). Peptides were either unphosphorylated, or phosphorylated at both ITIMs as follows: Dual phosphorylated peptide – FLUO- GRKKRRQRRRPQDLDQEPSLLY*ADLDHLALSRPRRLSTADPADASTIY*AVVV or unphosphorylated control peptide (lacking tyrosine phosphorylation) - FLUO- GRKKRRQRRRPQDLDQEPSLLYADLDHLALSRPRRLSTADPADASTIYAVVV. TAT sequences are underlined and phosphorylated tyrosines marked with an asterisk. Peptides were loaded into washed platelets by incubation with 10 µM peptide for 15 minutes at 30°C, after which they were assessed by light transmission aggregometry. Peptide loading conditions were optimised and verified by flow cytometry.

## Results

### G6B-b associates with both SHP-1 and SHP-2 in human platelets

**Figure 1:**
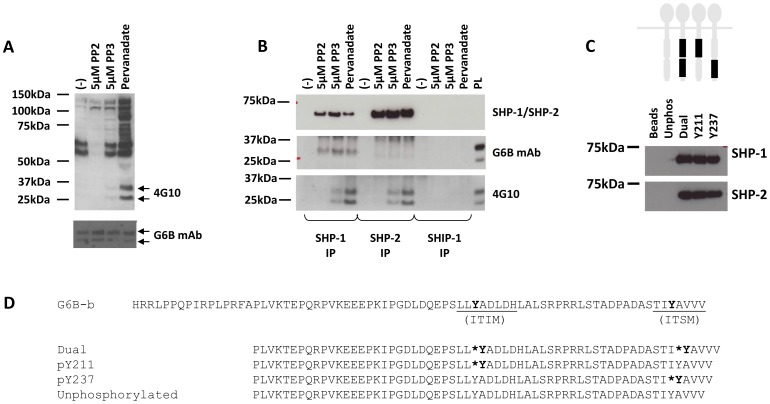
G6B-b binds to both SHP-1 and SHP-2 *in vitro*. (A) Human platelets were treated with PP2, PP3 or pervanadate to influence protein tyrosine phosphorylation as shown by 4G10 and G6B antibody staining. Arrows indicate G6B doublet. (B) G6B-b was co-immunoprecipitated from platelet lysates using anti-SHP1, SHP-2 or SHIP polyclonal antibodies (top panel). 4G10 staining revealed the presence of G6B-b (lower panel). (C). Immobilised peptides were added to pre-cleared lysates isolated from washed human platelets and incubated at 4°C for 30 minutes to allow peptide-protein associations to take place. Proteins were separated on 4–12% NuPage Bis-Tris gels and identified by Western Blotting (representative blots, n = 3). Black bars represent the position of the ITIM/ITSM motif in the receptor intracellular tails, either proximal or distal to the membrane. (D) Phosphopeptides (dual, pY211, pY237 or unphosphorylated) were synthesised corresponding to last 60 amino acids of the C-terminal intracellular tail (hG6B-b).

SHP-1 has been shown to associate with G6B-b in human platelets. SHP-2 has also been demonstrated to associate with G6B-b, but this has not been demonstrated in human platelets. We wanted to determine whether SHP-2 could associate with the receptor in human platelets as this interaction could relate to receptor function and regulation of GPVI signalling. As G6B-b is an orphan receptor, it is not possible to activate the receptor with its endogenous ligand. In the absence of receptor agonists, we carried out co-immunoprecipitation experiments in platelet lysates treated with the SFK inhibitor, PP2, its non-active analogue, PP3, or the tyrosine phosphatase inhibitor, pervanadate. This generated samples with G6B-b in a state of non-phosphorylation, partial/sub-maximal, or maximal phosphorylation, respectively (Figure 1A). Under these conditions, isolation of SHP-1 and SHP-2 by immunoprecipitation was robust (Figure 1B, top panel). Although our anti-G6B monoclonal was not sensitive enough to detect the receptor in immunoprecipitated material (Figure 1B, middle panel), detection of G6B-b with the anti-phosphotyrosine 4G10 antibody revealed the characteristic G6B-b doublet (Figure 1B, lower panel); an increase in the intensity of these bands is seen with increasing global cellular tyrosine phosphorylation. This data shows that G6B-b will co-precipitate with SHP-1 and SHP-2 in pervanadate-treated washed human platelets. No endogenous G6B-b was found to precipitate with the SH2 domain-containing inositol phosphatase, SHIP-1 (Figure 1B).

Previous mutagenesis experiments in Cos7 cells demonstrated that association of SHP-1 and SHP-2 with G6B-b relies on phosphorylation of the ITIM/ITSMs. Although likely that these interactions are direct, this has not yet been conclusively shown. In the absence of an endogenous ligand or receptor agonist, we used phosphopeptides corresponding to the intracellular portion of G6B-b in which the ITIM and ITSM reside. These biotinylated peptides were phosphorylated at either the ITIM, the ITSM, or at both sequences, to mimic receptor activation and phosphorylation. These peptides were incubated with platelet lysate and isolated using Streptacin beads. Peptide associated material was separated by SDS-PAGE and assessed for the presence of SHP-1 and SHP-2 by Western blotting, both of which were clearly identified (Figure 1C). Peptide sequences are summarised in Figure 1D. No SHP-1 or SHP-2 bound to the unphosphorylated control peptides.

### G6B-b directly interacts with the tyrosine phosphatases SHP-1 and SHP-2

**Figure 2:**
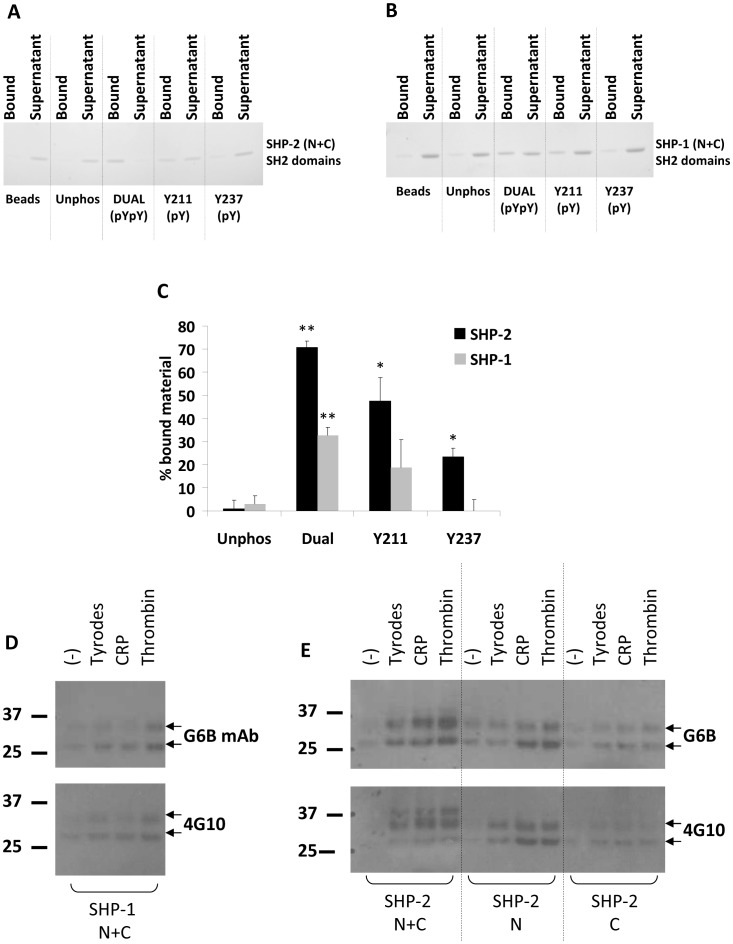
G6B-b ITIM/ITSMs show preferential binding to SHP-1 and SHP-2. Immobilised peptides were incubated with equimolar quantities of either SHP-2 (A) or SHP-1 (B) tandem SH2 domains. Unbound material was separated from the bound material and equal volumes loaded onto 4–12% NuPage Bis-Tris gels. Protein was visualised with Simply Blue Coomassie stain (Invtirogen). (C) Quantification of bound SH2 domain material to G6B-b phosphopeptides (n = 3, SEM, ** denotes p<0.01, * denotes p<0.05). G6B-b associates with SH2 domains from SHP-1 (D) and SHP-2 (E) in washed human platelet lysates activated for 90 s with either 1 µg/ml CRP or 0.5 U/ml thrombin.

Interactions between SH2 domains and ITAMs, ITIMs and ITSMs often rely on direct association between the phosphomotif-bearing receptor and its SH2 domain-containing binding partner. We wanted to verify that G6B-b was associating directly with SHP-1 and SHP-2 and not via an intermediary. To address this, we incubated G6B-b peptides with equimolar quantities of purified SH2 domain (either N or C or N+C) from SHP-1 and SHP-2. This approach revealed a robust association between SHP-2 and G6B-b phosphopeptides (Figure 2A and C) with ∼70% shifting from the unbound to the bound fraction for the dual phosphopeptide, and ∼50% and ∼25% binding to the Y211 and Y237 phosphopeptides, respectively. Only ∼30% of the tandem SH2 domains of SHP-1 bound to the dual phosphopeptide (Figure 2B and C) with little or no association with the individual ITIM or ITSM. These experiments indicate that the interaction between the G6B-b phosphopeptides and the SH2 domains of the tyrosine phosphatases is direct and also indicate that there is a difference in the recruitment of SHP-1 and SHP-2 to the receptor.

It is known that SH2 domains exhibit preferential interactions with different consensus phosphotyrosine sequences largely determined by the amino acids immediately downstream of the central phosphotyrosine [15]. Based on this study [15], the N-terminal SH2 domains of both SHP-1 and SHP-2 should preferentially bind to the ITIM (central pY at position 211) while the C-terminal domains should associate with the ITSM (pY at position 237). To determine whether this was the case and identify which interactions were most important for the interaction between the tyrosine phosphatases and G6B-b, we expressed the single SH2 domains (N or C) of SHP-1 and SHP-2. The N-terminal SH2 domain of SHP-1 was insoluble and it was not possible to purify sufficient quantities for binding studies. The C-terminal SHP-1 SH2 domain and both of the single SH2 domains of SHP-2 expressed well and monomeric material was purified. Pull down experiments were performed using purified SH2 domains of SHP-1 (N+C) and SHP-2 (N+C, N and C) to precipitate endogenous G6B-b from platelet lysate. As shown in Figure 2D, SHP-1 N+C SH2 domains were able to isolate endogenous G6B-b from platelet lysate. SHP-2 N+C SH2 domains (Figure 2E) were also able to isolate G6B-b from platelet lysate. In addition, the single N-terminal SH2 domain of SHP-2 also bound G6B-b while the C-terminal domain showed a very weak interaction.

**Figure 3:**
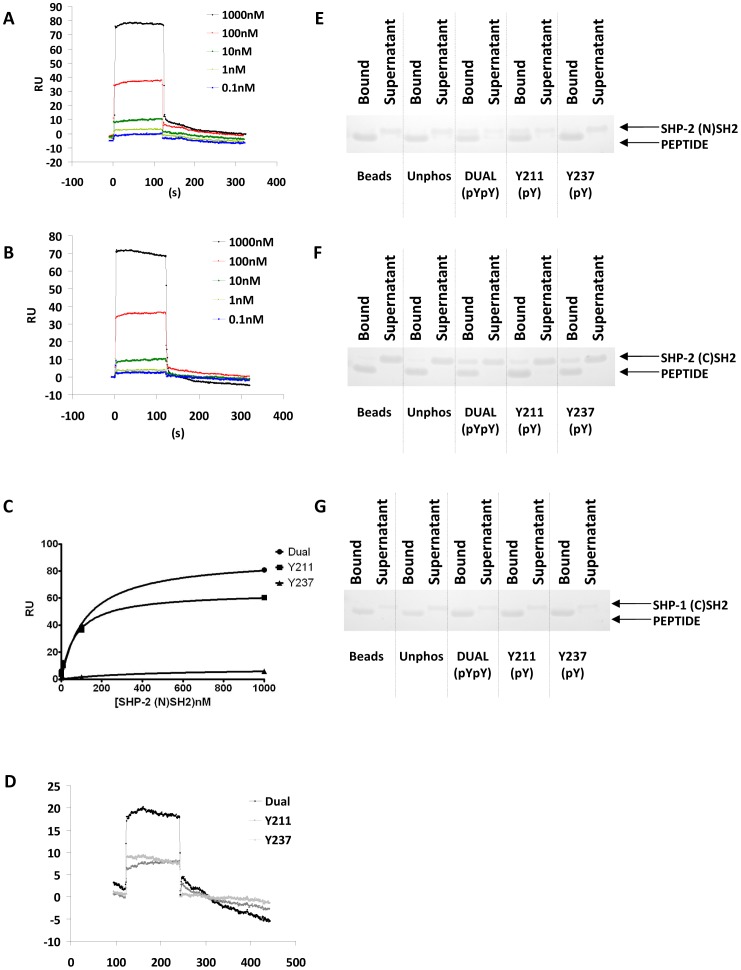
SHP-2 associates primarily through its N-terminal SH2 domain with G6B-b ITIM. The single SH2 domains of SHP-2 (N-terminal, ∼11 kDa or C-terminal, ∼13 kDa) or the C-terminal SH2 domain of SHP-1 (∼12 kDa), were injected over 50RU immobilised peptides. SHP-2 (N)SH2 bound well to both the dual (A) and Y211 (B) phosphopeptides (equilibrium binding is plotted in C), but not Y237 (data not shown). SHP-2 (C)SH2 exhibited minimal association with the phosphopeptides by SPR (D). Representative examples of direct binding assays for SHP-2 (N)SH2 (E), SHP-2 (C)SH2 (F) and SHP-1 (C)SH2 (G).

We next assessed these associations by surface plasmon resonance. The N-terminal SH2 domain of SHP-2 bound to both the dual (Figure 3A) and pY211 (Figure 3B) phosphopeptides. The dual G6B-b phosphopeptide bound the N-terminal SH2 domain of SHP-2 with a K_D_ of 130 nM (single site fitting). The pY211 peptide bound SHP-2 N(SH2) with a K_D_ of 110 nM (single site fitting). This was determined by plotting equilibrium binding (Figure 3C) as dissociation was too rapid to measure kinetically. Specific binding to pY237 was minimal; a K_D_ value of 10 µM was estimated by fixing maximum binding, suggesting that the interaction between the N-terminal SH2 domain of SHP-2 and G6B-b is primarily mediated through association with the Y211 ITIM. These data correlate well with a previous study which described the optimal binding site for SHP-2 N- and C-terminal SH2 domains [15]; the ITIM motif at position Y211 most closely resembles the consensus (N)SH2 sequence for SHP-2. According to this study, the C-terminal SH2 domain should bind to the ITSM at Y237 well, but we could only detect very weak binding of SHP-2 (C)SH2 by SPR (Figure 3D). Some association between the phosphopeptides and the N- and C-terminal SH2 domains could also be detected in the direct binding assay (Coomassie staining of bound and unbound material shown in Figure 3E and 3F) but this may be due to the higher concentration of peptide used in the direct binding assay and the difference in temperature (room temperature for direct binding assay verses 37°C for SPR). Despite the Y237 ITSM having an optimal binding site for the C-terminal SH2 domain of SHP-1 [15], no binding could be detected by SPR (data not shown) or *in vitro* binding assay (Figure 3G).

### SHP-2 has a higher binding affinity for G6B-b phosphopeptides than SHP-1

**Figure 4:**
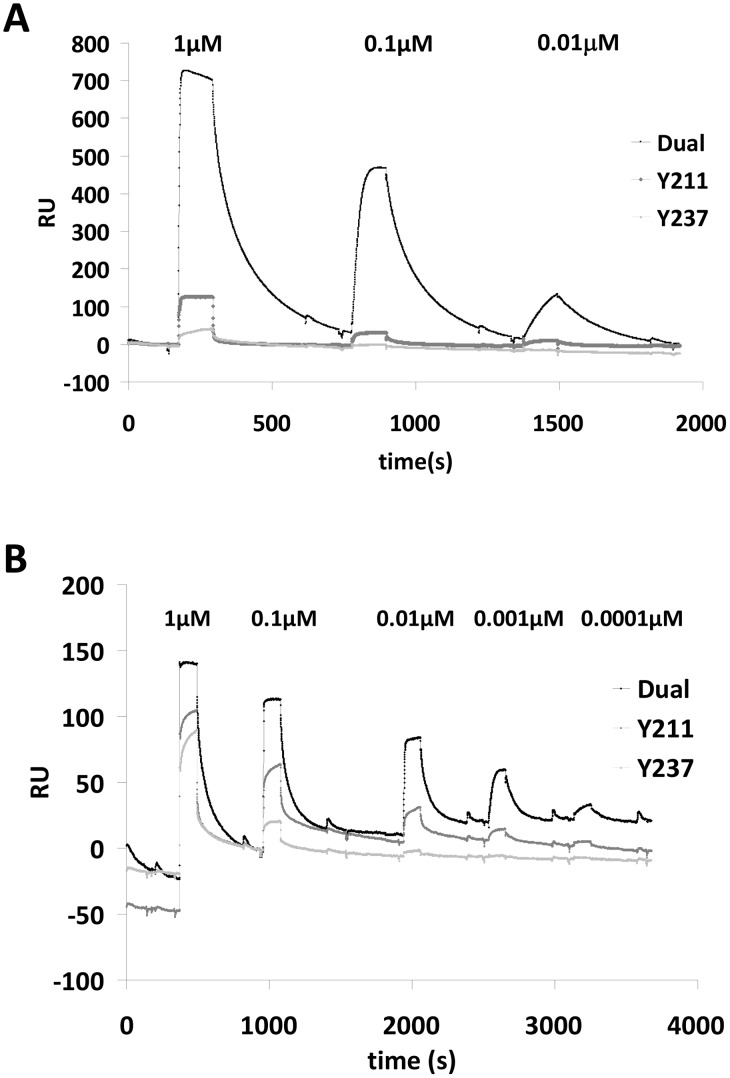
G6B-b has a higher binding affinity for SHP-2 than SHP-1. Purified tandem SH2 domains of SHP-2 (24 kDa) (A) and SHP-1 (68 kDa) (B) were flowed over 40RU immobilised peptide (7 kDa). Traces show specific binding to the dual (black line), pY211 (dark grey) or pY237 (light grey) phosphopeptides. Binding is enhanced by phosphorylation at both the ITIM and ITSM (dual peptide) for both SHP-1 and SHP-2 SH2 domains, compared to the single phosphomotifs. Data adjusted for non-specific binding to unphosphorylated peptide control. Representative traces, n = 3.

The data above all point to a role for both SHP-1 and SHP-2 in G6B-b signalling as both phosphatases can associate with the endogenous receptor, and with phosphopeptides *in vitro*. The interactions are direct and both *in vitro* binding experiments and SPR indicate that the G6B-b ITIM (Y211) is most important for receptor-phosphatase association with the second site playing a minor, but critical role. Previous studies have shown that using single phosphomotifs (isolated from sequences in which they may exist in tandem) and single SH2 domains can be misleading [8], [16]. We, therefore, assessed phosphatase-G6B-b interactions in more detail using the tandem SH2 domains of both SHP-2 (Figure 4A) and SHP-1 (Figure 4B). We kinetically determined the binding affinity (K_D_ = k_off_/k_on_) by SPR at physiological temperatures. The presence of both phosphomotifs dramatically enhanced association of SHP-1 tandem (N+C) SH2 domains to the dual phosphopeptide. Single site models were found to fit the data well while bivalent analyte models did not suggesting that SHP-1 tandem SH2 domains bind to the G6B-b dual phosphopeptide as a single binding unit, rather than as a two-site association, with an affinity of 35.3±4.3 nM (n = 5, SEM, Table 1).

**Table 1:** Association and disassociation constants for SHP-1 and SHP-2 binding to G6B-b phosphopeptides.

	K_on_	K_off_	K_D_
SHP-1 1000 nM	2.32×10^5^	0.00892	3.85×(10^−8^
SHP-1 100 nM	3.20×10^5^	0.01025	3.20×10^−8^
SHP-1 10 nM	5.63×10^5^	0.01141	2.03×10^−8^
SHP-1 1 nM	2.60×10^5^	0.01106	4.25×10^−8^
SHP-1 0.1 nM	2.40×10^5^	0.01040	4.33×10^−8^
SHP-2 1000 nM	1.67×10^7^	0.0106	6.34×10^−10^
SHP-2 100 nM	2.72×10^7^	0.0144	5.28×10^−10^
SHP-2 10 nM	1.41×10^8^	0.0206	1.45×10^−10^
SHP-2 1 nM	2.66×10^8^	0.1660	6.24×10^−10^
SHP-2 0.1 nM	1.52×10^8^	0.0720	4.74×10^−10^

K_on_ and K_off_ (s-1) were determined using Prism 5 software (GraphPad Software, California, U.S.A) at a variety of concentrations from Biacore 3000 runs carried out on three different days. K_d_ values = M.

SHP-2 tandem SH2 domains had a ∼100-fold higher binding affinity for the dual peptide than SHP-1 (K_off_ ∼0.01 s^−1^), with a K_D_ of 0.48±0.09 nM (n = 5, SEM, Table 1). Dissociation rates for SHP-2 were similar to that of SHP-1, but a higher association rate for SHP-2 (K_on_ = 1.21×10^8^±0.5×10^8^ s^−1^ for SHP-2 vs 2.23×10^5^±0.6×10^5^ s-1 for SHP-1, n = 5, SEM) was observed (Table 1). SHP-2 tandem (N+C) SH2 domains displayed enhanced binding to the dual phosphopeptide over the single phosphopeptides, but the effect was less pronounced than for SHP-1. Although SHP-2 can associate with the single phosphomotifs quite well, modelling suggests that the interaction between SHP-2 tandem SH2 domains and the dual phosphopeptide also most closely resembles that of a single site association. It should be noted that the binding affinity of SHP-2 tandem SH2 domains for the dual phosphopeptide was approximately 200-fold higher than that for the single N(SH2) domain, highlighting that although binding is mediated primarily through the (N)SH2 domain, the second domain dramatically increases the strength of the association.

### ‘Activating’ SH2 domain-containing proteins associate with G6B-b phosphomotifs

In this study we sought to determine a biochemical hierarchy for SHP-1 and SHP-2 recruitment to G6B-b, finding SHP-2 to have a higher binding affinity for the receptor than SHP-1. We also wanted to determine whether there were additional binding partners for G6B-b which could explain the observation that G6B-b can still inhibit GPVI signalling in the absence of SHP-1 and SHP-2. Using a method adapted from Hassan *et al* [17], Streptactin-Sepharose beads were coated with ∼5 nM of biotinylated phosphopeptide and incubated with platelet lysates. Associated material was screened for the presence of SH2 domain-containing proteins by Western blotting.

**Figure 5:**
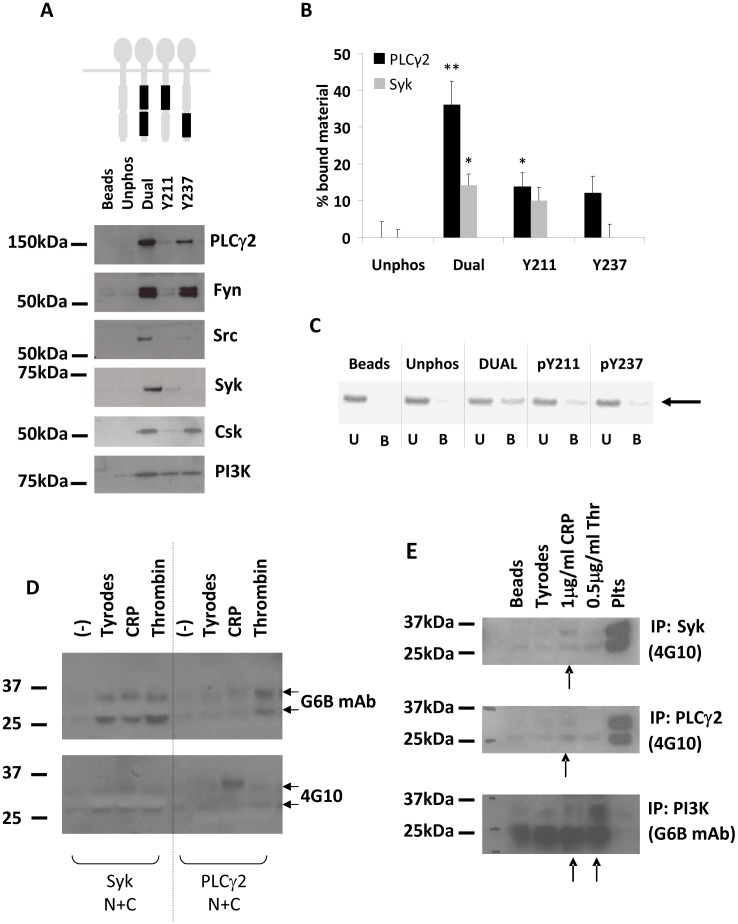
G6B-b associates with Syk and PLCγ2. (A) Peptide associated material was isolated from pre-cleared lysates separated on 4–12% NuPage Bis-Tris gels; proteins identified by Western Blotting (representative blots, n = 3). Black bars represent the position of the phospho-motif in the receptor intracellular tails. (B) Tandem SH2 domains for Syk and PLCγ2 were purified and incubated with equimolar quantities of G6B-b phosphopeptide. Bound material was separated from unbound material by SDS-PAGE and quantified (n = 3, SEM, ** denotes p<0.01, * denotes p<0.05). (C) Representative example of direct binding assay for PLCγ2. Arrow indicates PLCγ2 SH2 domains. (D) Purified SH2 domains (500 nM) of Syk or PLCγ2 were used to isolate G6B-b from platelet lysate stimulated with either 10 µg/ml CRP or 0.5 U/ml thrombin. (E) G6B-b was co-immunoprecipitated using Syk, PLCγ2 or PI3K polyclonal antibodies from platelets stimulated with 1 µg/ml CRP or 0.5 U/ml thrombin. Arrows indicate enhanced association.

A number of SH2 domain-containing proteins were found to associate with the G6B-b phosphopeptides *in vitro* including the SFKs Src and Fyn, PI3K, Csk, Syk and PLCγ2 (Fig. 5A). Association with the dual phosphopeptide was generally more robust; some preference for the pY237 (ITSM) over the pY211 (ITIM) was observed for PLCγ2, Csk, Src and Fyn. PLCγ2 was also identified as a potential binding partner by mass spectrometry from peptide-associated material isolated (data not shown) suggesting that it could interact with G6B-b in platelets. Further investigation using *in vitro* binding assays with purified SH2 domains revealed a direct association between G6B-b and both Syk and PLCγ2 SH2 domains. As shown in Figure 5B, around 40% of PLCγ2 N+C tandem SH2 domains were able to associate with the dual phosphopeptide while a small amount was found to associate with the Y211 phosphopeptide. A representative experiment showing Coomassie staining of bound and unbound material is shown in Figure 5C. Syk also showed a small but significant association with the dual phosphopeptide suggesting that these proteins have the potential to interact under physiological conditions. No association could be detected between G6B-b phosphopeptides and the SH2 domains of PI3K p85 or Csk using this approach. Endogenous interactions were also assessed by co-immunoprecipitation experiments using either recombinant N+C SH2 domains (Figure 5D) or antibodies specific for Syk, PLCγ2 or PI3K (Figure 5E). Association of Syk and PLCγ2 appears to be weak and could only be detected by 4G10 antibody binding. G6B-b could also be detected (using G6B monoclonal antibody) in material immunoprecipitated using anti-PI3K antibodies while no association could be detected for Csk.

**Figure 6:**
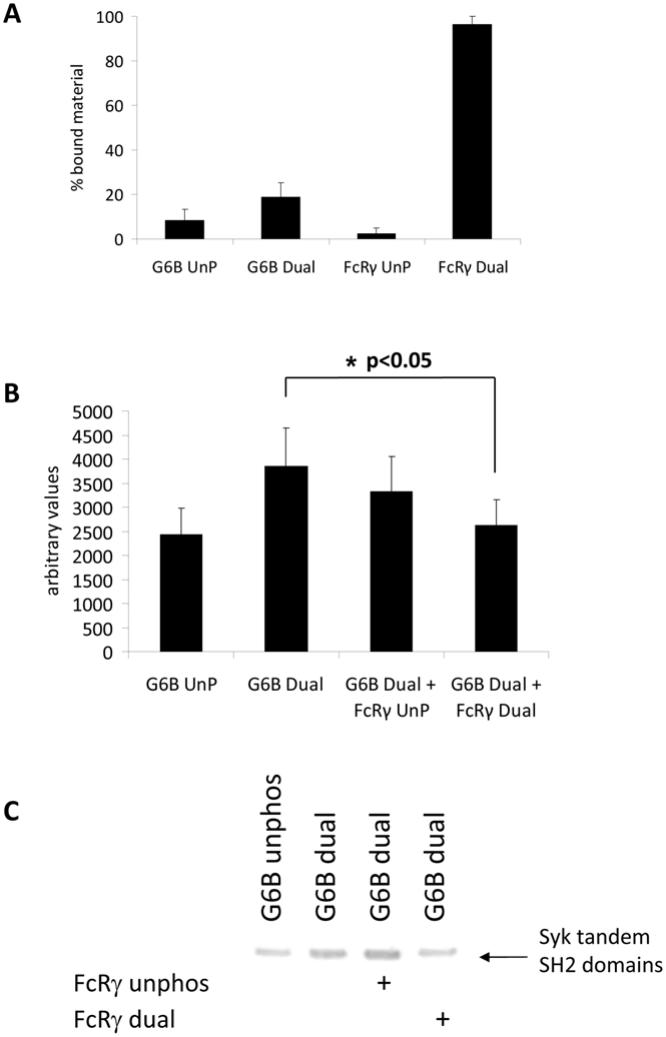
FcRγ can compete for Syk binding to G6B-b. (A) Syk binds weakly to G6B-b as shown by direct binding assay. Syk exhibits robust binding to FcRγ. (B) FcRγ can compete with phosphorylated G6B-b for Syk binding *in vitro*. Equimolar quantities of G6B-b and Syk were incubated in the presence of either unphosphorylated or dual phosphorylated FcRγ peptides. (C) Representative example of peptide competition by direct binding assay (arrow indicates Syk tandem (N+C) SH2 domains).

Despite the weak nature of these associations, it is possible that they could contribute to the SHP-independent inhibition of GPVI signalling by G6B-b observed in DT40 cells [11]. In the absence of SHP-1 and SHP-2, previously occupied binding sites will be available to associate with alternative binding partners and this could serve to restrict recruitment of Syk and PLCγ2 to their site of action. Because Syk is known to bind to the FcRγ chain and initiate tyrosine kinase signalling from the GPVI receptor, perturbation of this association would directly inhibit signalling from the collagen receptor. We wanted to see whether the phosphorylated FcRγ chain could compete with phosphorylated G6B-b for Syk binding. We first assessed association of Syk tandem SH2 domains with both G6B-b and FcRγ phosphopeptides (Figure 6A). Nearly all the Syk N+C(SH2) (∼95%) binds to the phosphorylated FcRγ while only a small amount of Syk associates with the G6B-b dual peptide (∼20%). In the presence of FcRγ dual phosphopeptide, binding of Syk tandem SH2 domains to the G6B-b dual phosphopeptide is reduced to that of the unphosphorylated peptide (Figure 6B and C) indicating that phosphorylated FcRγ can compete for Syk binding. Again, these associations are weak and unlikely to occur in the presence of other G6B-b binding partners, such as SHP-1 and SHP-2, but in the absence of endogenous binding partners, these associations are possible.

**Figure 7:**
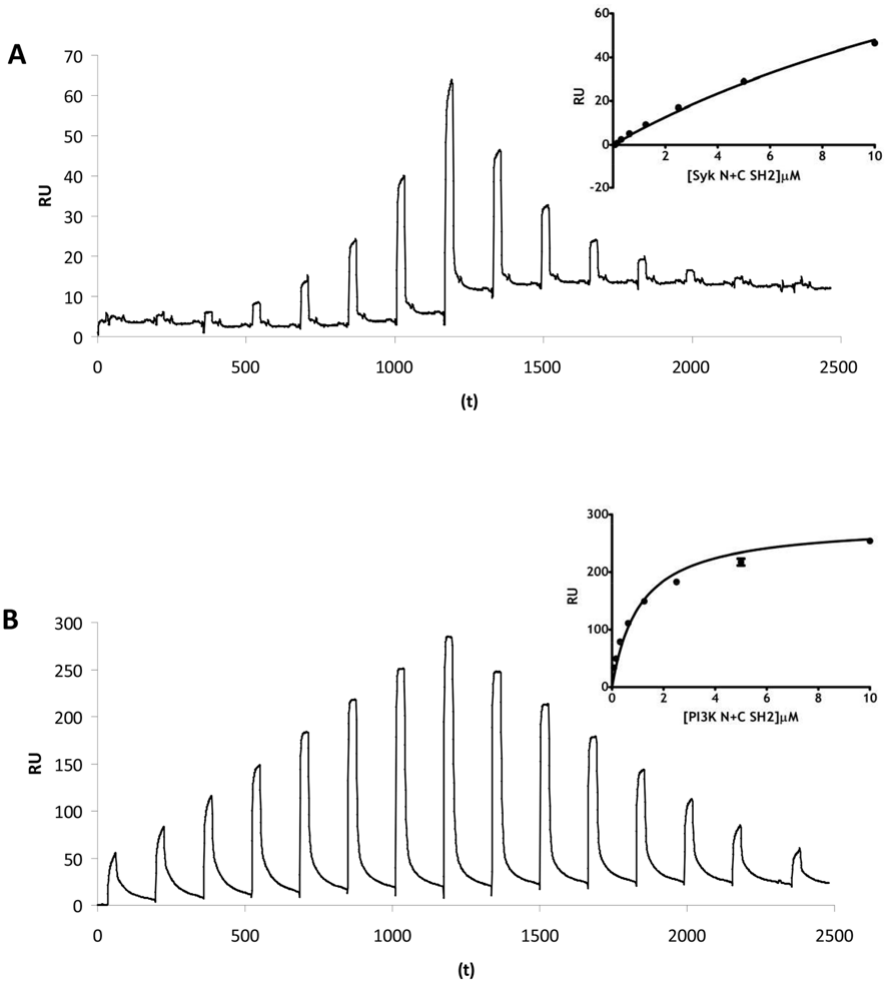
G6B-b can associate with both Syk and the regulatory subunit of PI3K. (A) Representative SPR trace demonstrating Syk/G6B-b association. Inset shows summary of binding data (n = 3, SEM). (B) Representative trace for PI3K p85 SH2 domain association with G6B-b phosphopeptides by SPR. Inset shows data summary (n = 3, SEM).

As we had purified SH2 domain material, we also used SPR to measure protein∶peptide interactions. Although PLCγ2 and Src bound in the *in vitro* binding assay, we could not detect any association by SPR. Syk was found to bind very weakly to the dual peptide (K_D_∼25 µM, Figure 7A), while PI3K p85 SH2 domains bound with a K_D_ of ∼1 µM (Figure 7B).

**Figure 8:**
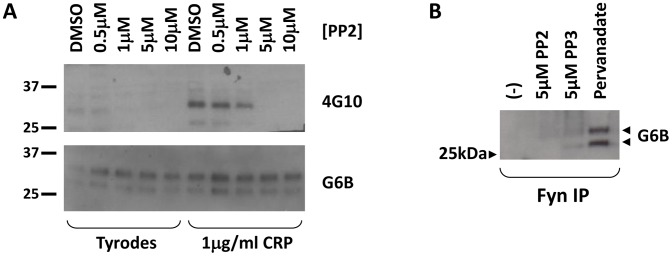
G6B-b is phosphorylated by Src family kinases. (A) Washed human platelets were incubated under stirring, but non-aggregating, conditions with the SFK inhibitor, PP2, or DMSO, prior to the addition of 1 µg/ml CRP or Tyrodes buffer. Samples were collected after 5 minutes. Tyrosine phosphorylation was detected by 4G10 staining (top panel, n = 4). Bottom panel shows G6B-b protein levels to confirm equal loading. (B) G6B-b co-immunoprecipitates with Fyn in pervanadate-treated washed human platelets.

Finally, based on previous reports of their role in both PECAM-1 [18] and GPVI [19] phosphorylation, it is likely that SFKs are responsible for phosphorylation of G6B-b. Indeed, inhibition of SFK activity with the inhibitor PP2 reduced the G6B-b tyrosine phosphorylation in response to CRP (Figure 8). The inactive PP2 analogue, PP3, had no effect on tyrosine phosphorylation (data not shown). As previously mentioned, both Src and Fyn were identified in the pull down assays as able to associate with G6B-b peptides. Phosphorylated G6B-b was detectable by the 4G10 antibody on material isolated by endogenous co-immunoprecipitation using anti-Fyn antibodies, but not with anti-Src antibodies. In addition, soluble Src SH2 was unable to associate with the dual phosphopeptide by SPR, suggesting that Fyn is the most likely SFK candidate to phosphorylate G6B-b in both resting and activated cells.

### G6B-b ITIM motifs influence cell sensitivity to CRP

**Figure 9:**
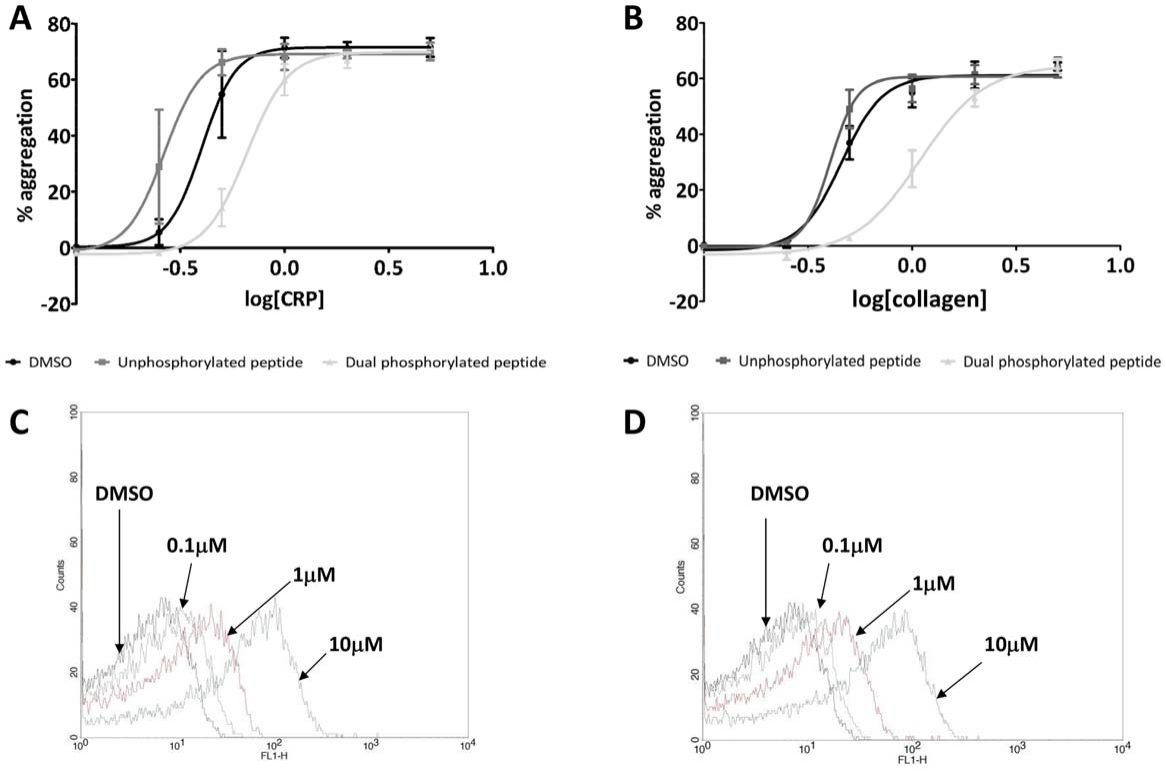
TAT-mediated transduction of G6B-b phosphopeptides reduces platelet sensitivity to GPVI agonists. Washed human platelets were loaded with FITC-conjugated, TAT-labelled phosphopeptides, prior to assessment of aggregation responses to CRP (A) or collagen (B). Control responses (DMSO control) is shown by the black line, unphosphorylated peptide is in dark grey while phosphorylated G6B-b peptides are represented by the light grey line. To demonstrate loading, platelets were incubated with peptide for 15 minutes at 30°C before being analysed for fluorescence by flow cytometry. Representative experiments are shown for the unphosphorylated control peptide (C) and the dual phosphopeptide (D).

Based on the cell-specific expression and relatively high copy number of G6B-b, it is likely that this receptor is playing an important role in the regulation of platelet activity. From the work shown above, G6B-b has the potential to associate with a number of SH2 domain-containing proteins, with the major binding partners being SHP-1 and SHP-2. As platelets are anucleate and no transgenic model is currently available with which to study G6B-b function, we decided to use a protein transduction approach [20]–[22] to load human platelets with G6B-b peptides and look for changes in cell responses to agonist. Washed human platelets were loaded with TAT-G6B-b peptides (either dual phosphorylated or unphosphorylated) and changes in CRP efficacy determined by light transmission aggregometry. As can be seen from Figure 9A, transduction of the dual phosphorylated G6B-b TAT-peptide increases the EC_50_ of CRP compared to the vehicle control (from 0.4 µg/ml to 0.65 µg/ml, representing a ∼1.65-fold increase), therefore reducing platelet sensitivity to agonist. Effects on the endogenous GPVI receptor ligand, collagen, were more pronounced (Figure 9B), with a 2.5-fold increase in EC_50_ from 0.4 µg/ml to 1 µg/ml. Thrombin-mediated activation was unaffected (data not shown). Increasing the concentration of G6B-b dual phosphorylated peptide inside the cell reduces platelet sensitivity to GPVI agonists. Peptide loading was verified by flow cytometry (Figure 9C and 9D.

## Discussion

Platelet activation is a tightly regulated process, being influenced by a combination of both positive and negative signals. The ITIM/ITSM-bearing receptors PECAM-1, CEACAM1L and G6B-b can all inhibit platelet function and have been shown to associate with SH2 domain-containing phosphatases. Our data revealed differences in the binding properties of SHP-1 and SHP-2 for G6B-b. While both phosphatases have the same dissociation rate for the dual phosphorylated peptide (K_off_ = 0.01 s^−1^), SHP-2 has a more rapid association rate, resulting in a higher affinity for G6B-b than SHP-1. SHP-2 tandem SH2 domains bind to the G6B-b dual phosphopeptide with an affinity (K_D_ = 0.48±0.09 nM) similar to that previously reported for an optimal SHP-2 binding protein, SHPS-1/SIRPα [23]. As this phosphatase is generally associated with cell activation, this was a surprising finding. However, a preference for SHP-2 binding over SHP-1 has previously been observed for PECAM-1 [24]–[26], and this phosphatase is also recruited to TLT-1, an activating, ITIM-bearing platelet receptor [27]–[29]. Although SHP-2 binding is maximal when both G6B-b phosphomotifs are phosphorylated, this phosphatase, unlike SHP-1, can associate with either of the single phosphomotifs; SHP-2 seems less constrained in terms of its requirements for binding. The ∼100-fold higher binding affinity of SHP-2 for G6B-b may dominate to form an inhibitory pathway that maintains the cell in a resting state alternative to, or in addition to, that employed by SHP-1. It is not yet clear what role SHP-2 is playing in G6B-b-mediated inhibition of platelet activation but SHP-2 has been reported to bind to other ITIM/ITSM receptors [30], [31] to inhibit cell activation. One example has recently been proposed for PECAM-1-mediated inhibition of GPVI signalling in platelets. SHP-2 is recruited to the LAT signalosome in an ‘activating’ capacity, where it assists in PI3K recruitment via its p85 regulatory subunit. This SHP2/p85 inteaction therefore serves to promote recruitment of PI3K to a substrate-rich environment. When PECAM-1 is phosphorylated, it essentially competes for this complex, depleting p85/SHP2 from the LAT signalosome and therefore, GPVI-mediated cell activation [32]. It is possible that G6B-b acts in a similar way to PECAM-1 by inhibiting cell activation through a redistribution of the signalling molecules. While PECAM-1 recruits p85 via SHP-2, we found that G6B-b can directly interact with p85 (with a K_d_ of ∼1 µM) and SHP-2 (Kd = 0.5 nM), independently. Although the G6B-b/p85 interaction is much lower than the nanomolar binding affinities documented for other p85-binding proteins, SPR showed this interaction to be direct and within physiological limits for SH2-pY associations.

Although both G6B-b and PECAM-1 are both inhibitory ITIM-bearing Ig-like platelet receptors, they vary in a number of important ways. In terms of extracellular characteristics, G6B-b has a much smaller extracellular domain than PECAM-1 such that its exclusion at the platelet synapse is less likely, and its expression at the cell surface is enhanced by platelet activation while PECAM-1 is shed from the cell surface. In terms of intracellular effects, PECAM-1 inhibits Ca^2+^ release [33] while G6B-b does not [1]. Some overlap in binding partners is observed for these receptors yet they also show some specifity for intracellular binding partners. It is likely that these receptors have some specific as well as overlapping roles in the regulation of platelet activity. They do both recruit SHP-2 and they may do so for similar reasons, but not in the same way. Previous studies have shown that SHP-1 and SHP-2 associate with PECAM-1 ITIMs with affinities of between ∼60–350 nM, which is lower than that for G6B-b [34]. However, these studies were carried out using single, rather than the tandem, phosphomotifs. Determination of the binding affinities of other inhibitory receptor∶protein interactions will help put G6B-b associations into a physiological context relative to the other receptors present at the cell surface.

In this study we also detected a number of other SH2 domain-containing proteins in phosphopeptide-associated material. In addition to the p85 regulatory subunit of PI3K, Syk and PLCγ2 were found to weakly associate with endogenous G6B-b in pervanadate-treated cells, but these interactions appear somewhat tenuous and secondary to the interactions of G6B-b with its dominant binding partners, SHP-1 and SHP-2. Mori *et al* [11] recently demonstrated that G6B-b was still able to attenuate CLEC-2 and GPVI signalling in DT40 cells deficient in both SHP-1 and SHP-2. This result may be explained by the fact that G6B-b has the potential to recruit alternative binding partners in the absence of SHP-1 and SHP-2, and that these interactions may explain the observation reported in the Mori *et al* (11) study. Although the affinity for activating binding partners is weak, G6B-b is present at a high copy number at the cell surface [4]; in the case of GPVI/FcRγ, G6B outnumbers the GPVI receptor by more than 20∶1. In the case of Syk, G6B-b is also partially phosphorylated under resting conditions and as such, can compete for any available Syk SH2 domains until sufficient FcRγ chain is phosphorylated to overcome these interactions and dominate Syk SH2 domain occupancy.

It is likely that G6B-b contributes to platelet regulation by employing both passive (via interactions with activating proteins such as Syk and PI3K to prevent spurious cell activation) and active (recruitment of SHP-1 and SHP-2 to the membrane) mechanisms. One might envisage a situation where in the resting cell, G6B-b is able to associate with any number of SH2 domain-containing proteins, including Syk, PLCγ2 and p85, such that it can act as a sink, or buffer, for these proteins. Once the cell is activated by collagen, FcRγ will be phosphorylated, generating a high affinity binding site for Syk, which will then predominantly associate with the ITAM to initiate cell signalling. This then leads to LAT phosphorylation and subsequent recruitment of PI3K. Although this hypothesis is highly speculative, some evidence to support this theory is presented in the peptide-loading experiments described above. Loading dual phosphorylated peptide into washed human platelets reduces cell sensitivity to GPVI ligands; if G6B-b was acting as a ‘sink’ for activating SH2 domain-containing proteins, one would predict that an excess of phosphopeptide would increase the number of alternative binding sites for these proteins and therefore increase the EC_50_ of GPVI agonists. It is also possible that the presence of G6B-b phosphopeptide would compete for SHP-1 and SHP-2 binding, impeding their recruitment to the membrane. If SHP-1 and SHP-2 have inhibitory roles, one might expect that their loss from signalling hubs close to the membrane would increase cell sensitivity to agonist but this does not appear to be the case. The lack of an endogenous ligand for G6B-b has been limiting with regards to elucidating its role in cell activation and function, and it is therefore difficult to progress this work and fully characterise the receptor. Despite this, we have identified a number of alternative binding partners for G6B-b that may shed light on the work by Mori *et al* [11], and have generated data that points to a potentially non-canonical role for G6B-b in platelet function, rather than a solely basic inhibitory role.

Receptor-ligand dynamics will influence cell activity and are themselves influenced by copy number, localisation, phosphorylation and binding affinity. The combination of all these factors will serve to buffer spurious cell signalling events that could lead to inappropriate platelet activation and thrombus formation *in vivo* and ensure that cell activation occurs only in the presence of an appropriate physiological stimulus.
